# Advanced quantum image representation and compression using a DCT-EFRQI approach

**DOI:** 10.1038/s41598-023-30575-2

**Published:** 2023-03-13

**Authors:** Md Ershadul Haque, Manoranjan Paul, Anwaar Ulhaq, Tanmoy Debnath

**Affiliations:** School of Computing Mathematics and Engineering, Charles Sturt University, Bathurst, NSW 2795 Australia

**Keywords:** Quantum information, Quantum optics

## Abstract

In recent years, quantum image computing draws a lot of attention due to storing and processing image data faster compared to classical computers. A number of approaches have been proposed to represent the quantum image inside a quantum computer. Representing and compressing medium and big-size images inside the quantum computer is still challenging. To address this issue, we have proposed a block-wise DCT-EFRQI (Direct Cosine Transform Efficient Flexible Representation of Quantum Image) approach to represent and compress the gray-scale image efficiently to save computational time and reduce the quantum bits (qubits) for the state preparation. In this work, we have demonstrated the capability of block-wise DCT and DWT transformation inside the quantum domain to investigate their relative performances. The Quirk simulation tool is used to design the corresponding quantum image circuit. In the proposed DCT-EFRQI approach, a total of 17 qubits are used to represent the coefficients, the connection between coefficients and state (i.e., auxiliary), and their position for representing and compressing grayscale images inside a quantum computer. Among those, 8 qubits are used to map the coefficient values and the rest are used to generate the corresponding coefficient XY-coordinate position including one auxiliary qubit. Theoretical analysis and experimental results show that the proposed DCT-EFRQI scheme provides better representation and compression compared to DCT-GQIR, DWT-GQIR, and DWT-EFRQI in terms of rate-distortion performance.

## Introduction

In the field of quantum, computer science, physics, and mathematics play vital roles in concrete quantum information processing (QIP)^1^. Hilbert space provides plenty of space to map quantum information in the quantum domain. In a Hilbert space, the state of the quantum mechanics is described by the quantum vector. In a quantum system, quantum mechanics mainly deals with quantum properties. Entanglement and superposition are two main properties in quantum mechanics that provide faster computation^2^. When an image is represented in a multi-particle quantum than the image is known as a quantum image. In the quantum image, qubits replace the classical bits in an array of pixels and show better the better reproduction of original stored values compared with the classical approach’s (i.e., stochastic)^3^. Nowadays, due to more rapid analysis and remarkable quantum properties, it has gained more research interest worldwide. On the other hand, quantum parallelism is the inherent phenomenon that makes it unique and proven faster than classical computers^4,5^. The limitations of classical computers are given below^6,7^.

- Unable to solve NP (non-deterministic polynomial)-hard problems rapidly.
- Finding the pattern work is completely routine and requires no details understanding of the subject of the problem.
- Slow computational time compared to a quantum computer.
- Optimization–Optimization is finding out the best solution to a problem among many possibilities.

According to Moore’s law, the computing power of classical computers has increased in the past decade. After that, it’s computing power has not increased significantly due to the limitation of several objective factors^8^. Therefore, it is the demand of time, to increase the computing power. Feynman et al. explored the first quantum computer another way to increase computing power which attained popularity in the research and development community^9^. In^10^, Shor proposed an algorithm for factorial calculation for integers in quantum computers, which showed faster computation than classical computers. After that, following the Shor algorithm, Grover provided an algorithm for database search in quantum domain^11^. The advantages of quantum computers compared to classical computers are given below^6,7,12,13^.

- It can rapidly solve NP problems.
- In terms of hardware, its capable to make every possible processing answer simultaneously.
- Faster computational time than a classical computer
- Can carry an enormous amount of information on account of advantages of exponential formula $(2^n)$.
- Able to create mathematical creativity automatically.
- Dramatically increase the speedup in case of cryptographic codes and internet base monetary transactions.

However, there are still some limitations of quantum computer^6,7,12,13^ which are listed below:

- For other applications, such as chess playing, airline flight schedules, and providing theorem, the quantum computer still suffers the same algorithmic limitation as today’s classical computer.
- Generate error due to unwanted interaction between the quantum computer and its environment, typically known as decoherence.
- Unfortunately, there is a catch. During measurement, it can display only one result among many possibilities. All the other results will then disappear.
- As transistors in microchips approach the atomic scale, ideas from quantum computing are likely to become relevant for classical computing as well.

Although there have some pros and cons, still there are a lot of areas of application and development of the quantum computer. The areas of application of a quantum computer are the identification of pattern recognization in the stock market, recording of weather or brain activity, drug discovery process, cryptographic systems, chemical simulation, radar making, and weather forecasting^7,13^. Applications of a quantum computer in terms of image processing are image representation, image compression, edge detection, security, and denoising of images, information security, watermarking, high privacy, QCNN (Quantum Convolutional Neural Network)^6,14^.

Moreover, representing and compressing the classical image into the quantum domain is still challenging due to the high complexity of the preparation. To address the presentation and compression issue of a classical image inside the quantum computer gradually accelerates the combined application of quantum computers and classical computers^8^. In many applications, image processing plays core characteristics of image operation^15^. In classical image processing, the number of operations required is relatively high. The application of the image area is increasing daily, such as image pattern learning, image registration, image sensor data, agriculture, medicine, and remote sensing. Moreover, the number of processing images becomes bulky leading to the algorithm’s complexity in classical computation. In classical computation, processing those huge images requires more memory and hardware^16^.

In classical image computing, images are defined as metrics numbers that represent the color or intensity of every pixel in a Cartesian coordinate system with its location. In classical image processing, normally spatial correlation is exploited within implicit consideration of the location of the pixel as those are always associated with pixel intensities. However, in quantum image computing, we need to explicitly encode the pixel intensities and the corresponding locations using qubits. In the pixel intensities, we only consider ‘1’ position of the binary representation. Sometimes, we also convert transform coefficients or quantized transformed coefficients instead of pixel intensities for quantum image computing. The traditional image-processing approaches require higher computational resources to process and store the images.

Quantum computing (QC) in image processing provides a quadratic speedup compared to classical computation^17^. In QC, a qubit or quantum bit is the basic unit of quantum information. The quantum version of the classical binary bit is physically realized with a two-state device. In 2003, Venegas-Andraca and Bose proposed a method called Qubit lattice to map the four different random pixel values into single qubits^3^. In QC, FRQI (Flexible Representation of Quantum Image) is the first approach that represents and encodes the binary classical image into the quantum domain based on the probabilistic representation of amplitude^18^. It represents the image pixel values in the quantum domain using the control rotation matrices. The control rotational matrices can be implemented using a c-not gate and standard rotation. The NEQR is another approach that represents and stores the pixels of grayscale images in a normalized superposition^19^. It is a deterministic approach, not probabilistic^20^. In NEQR, after converting the pixel values into a binary system, only the frequent number of ones is considered to map the pixel value in a quantum system. The pixel and state (position) preparation qubits are used to map pixel values and their corresponding state position of the pixel.

As mentioned earlier that the traditional image-processing approaches require higher computational resources to process and store the images. Therefore, it is necessary to find high-performance algorithmic support for computing platforms to process and store a high amount of image data^21^. To address high-performance image processing computing, quantum is the right candidate, which operates on a qubit rather than a binary bit^3^. The complexity of the quantum computer for the n-bit sequence is *O*(*n*), whereas the classical computer requires $O(n*2^n)$. Quantum image compression reduces the number of image operations to prepare the quantum image in the quantum domain. The number of gates is the primary resource in a quantum system to determine its complexity rather than counting qubit. Moreover, the total number of required gates determines the quantum network time complexity, where each needs a certain time to do the operation^22^. In computing power, storage, and communications, continuing cost improvements are making more and more systems practical and compression is almost always included to provide cost-effective solutions.

A DCT-EFRQI approach is proposed for quantum image representation and compression through the classical preparation method and compares the result with an existing method to measure the proposed approach’s performance. Generally, the FRQI approach uses rotating gates to store grayscale pixel values in the probabilistic-based amplitude mapping in the Bloch sphere. However, in this article, the proposed DCT-EFRQI approach is based on the NEQR model which is deal with the coefficient value of the pixel rather than a direct pixel. DWT(Daubechies Wavelet Transform) and DCT are used to prepare the image before presenting it in the quantum system to investigate their capability for the preparation process. Then, quantized the coefficient values to make it feasible to represent and compress in the quantum circuit. After that, the quantized coefficient is represented in the quantum computer using the proposed algorithm. Before representing in a quantum computer, the quantized coefficient and its corresponding position are prepared to make it appropriate for quantum representation and compression.

The proposed DCT-EFRQI approach can be a lossy or lossless compression technique where the transmission channel cannot transmit information due to its limited capacity. If we use quantization after applying DCT, then it can be lossy compression so that we can reduce the required bits based on the channel capacity with the different labels of quantizations, obviously, this also sacrifices the quality of the image. Through transformation and quantization, we try to keep important information in such a way that the visual impact of the image quality is not significant. Therefore, the proposed method is applicable where we need to reduce the required bits without significantly sacrificing image quality.

The contributions of this work are given below.

- Represent and compress the different sizes of images in a quantum computer.
- Preparation complexity is removed due to applying the preparation approaches.
- The Quantum bit can be calculated for any size of the image.
- Image can be retrieved from its quantum circuit, including pixel and position.
- Any quantum operation can be included through our approach.
- Things are happening in quantum computers, but resource calculation is done through classical computers.
- Combining applications bring the real-life quantum image application.

The rest of the article is organized as follows. The literature survey is discussed in section “Literature and its summary”; the proposed methodology is presented in section “Proposed methodology”; the result and discussion are given in section “Result and discussion”. The conclusion of this work is outlined in section “Conclusion”.

## Literature and its summary

Inspired by the pixel representation of an image in classical computers, FRQI (Flexible Representation of Quantum Image) was developed^20^. The FRQI represents the gray scale image information as an angle in the quantum system. FRQI cannot represent pixel-wise grayscale complex operation since it used only one qubit. The Entanglement image representation was proposed in 2010 to take advantage of entanglement use^18^. Based on JPEG (Joint Photography Expert Group) image, Jiang et al. proposed an efficient quantum compression method using the GQIR approach that used the DCT preparation method in 2017^23^. It uses DCT and GQIR approaches together to increase the compression known as DCT-GQIR.

On the other hand, both DCT and DWT are the two effective and popular transformation techniques that are normally used for image or video compression because of their excellent capacity to concentrate different frequency information in different locations. In this approach, our objective is to understand which transformation is more effective to represent and compress images in the quantum domain. Thus, we have performed experiments using various quantization levels, block sizes, and quantum conversion techniques to evaluate the representation and compression ability.

Generally, the DCT transforms decorrelate the information based on the frequency. For a 2D DCT transformation, the top-left corner has low-frequency information, and the bottom-right corner has a high frequency. As the human visual system is sensitive to low-frequency information, thus, we can use quantization to discard high-frequency information. In the proposed method we have applied DCT in the 8 × 8 size block^24^. We also add bits information in the bit rate calculation as the location of each block is different. After applying DCT and then transferring the coefficient into the GQIR approach and both together are known as DCT-GQIR.

In addition, the DWT is also considered to take the advantage of the image compression with GQIR known as the DWT-GQIR approach. In the DWT approach, the wavelet is sampled at the discrete interval. It provides both frequency and spatial domain information about the image. In each decomposition label, it uses pair of low and high pass filters. The approximate and detailed information of the image is extracted by the high and low pass filters respectively. The 2D DWT decomposes the image into four sub-band; the top-left corner contains the low-frequency component (LL) in the horizontal and vertical position, the bottom-right corner contains the high-frequency component in the horizontal and vertical position, the top-right corner contains the high and low-frequency component in the horizontal and vertical position respectively, the bottom-left corner contains the low and high frequency in the horizontal and vertical position respectively^25^. The preparation approach is the main difference between DCT-GQIR and DWT-GQIR approaches.

**Figure 1:**
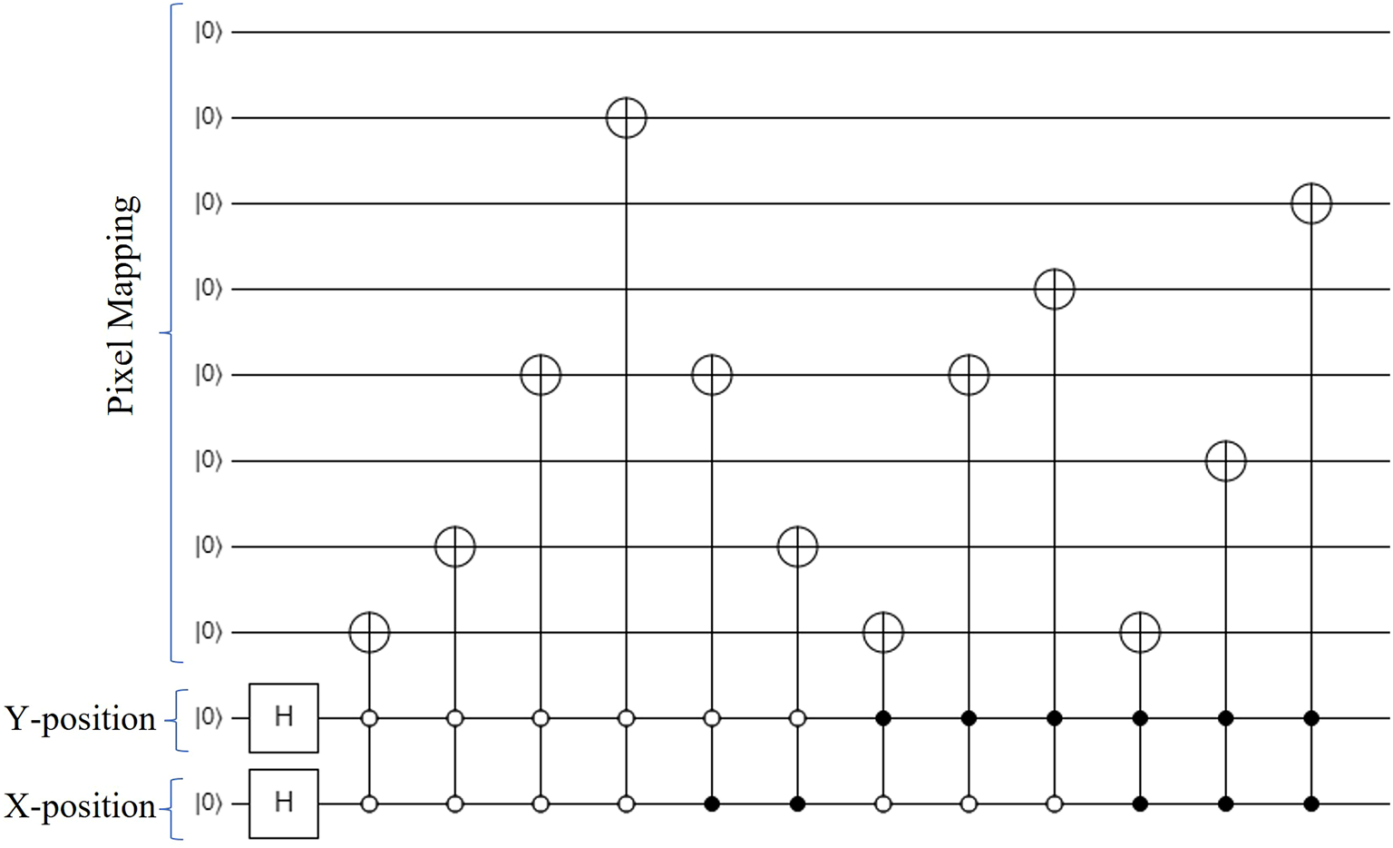
Quantum circuit for the GQIR representation of a $2\times 2$ gray scale image.

Laurel et al. proposed a quantum-based equivalence pixel image from a bit pixel image^26^. The NEQR (Novel Quantum Representation of Color Digital Images) was proposed to represent the classical color image in a quantum system^19^. A NEQR method resolves the FRQI issue because it provides several qubits to represent grayscale images. The limitation of NEQR is that it can only represent the square image. To solve this problem, the INEQR approach was proposed to represent unequal horizontal and vertical length images^21^. In INEQR, how color and big-size images are represented is still unclear. Author Li et al. proposed a GNEQR approach in 2019 that uses $n+8+2$ qubits representing a 24-bit RGB color image with $2^n$ pixels^27^. A multilevel 2-D Daubechies quantum wavelet transform (QWT) has been proposed in 2022 for quadratic speedup of quantum image computation^28^. The GQIR (General Quantum Image Representation) uses a logarithmic scale to represent a rectangular image of arbitrary shape but generates many redundant bits for position preparation^29^. Figure 1 exhibits the corresponding $2\times 2$ GQRI quantum circuit representation of an image whose pixel values are $79(X=1, Y=1)$,$10(X=1, Y=2)$, $25(X=2, Y=1)$, and $37(X=2, Y=2)$ respectively. It directly converts the pixel value and its position into the quantum circuit. For each pixel position connection, every time a similar amount of bits is required to connect each cnot gate for preparing pixel value with its state position is the main drawback of this method. Actually, it’s developed from FRQI which represents the color and location information of the image. Its maps the qubits in the vertical and horizontal coordinate system where the logarithm function is used to represent the original image {H*W}, where, $H=\lceil log_2H \rceil$ and $W=\lceil log_2 W \rceil$. Both color and location information are normalized in the $\vert 0 \rangle$ and $\vert 1 \rangle$ qubits. $\vert XY \rangle$ is the location information and the color information. It needs $(h + w + q)$ qubits to represent a $H\times W$ image with gray range 2*q*. Note that GQIR can represent not only grayscale images but also color images because the color depth *q* is a variable. In most cases, when $q=2$, it is a binary image; when $q = 8$, it is a grayscale image; and when $q = 24$, it is a color image. For position encoding, the number requires qubits is calculated from the maximum size of the image. For example, for $512\times 512$ image, the number of required qubits for encoding Y and X positions are 9, and 9 qubits respectively.

**Figure 2:**
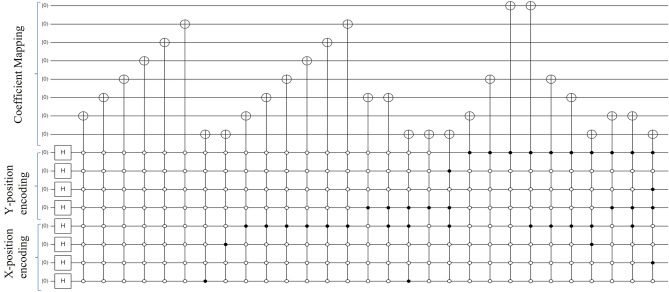
A $16\times 16$ DCT-GQIR quantum image including state preparation.

In practical application, Figure 2 shows a $16\times 16$ deer image quantum circuit as an example after performing the DCT preparation approach and 70 quantization factors using the Quirk simulation tool^30^. The coefficient values of a $16\times 16$ deer image are $126(X=1,Y=1)$, $1(X=1,Y=9)$, $4(X=2, Y=1)$, $4(X=2, Y=9)$, $1(X=2, Y=10)$, $1(X=4, Y=1)$, $1(X=6,Y=9)$, $138(X=9,Y=1)$, $140(X=9,Y=9)$, $2(X=10,Y=1)$,$2(X=10,Y=9)$ and $1(X=11,Y=9)$ respectively. To prepare the coefficient value in the quantum domain, first, convert the coefficient of nonzero values into binary and then maps the corresponding ones only using 8 numbers of qubits shown at the top of the quantum circuit in Fig. 2. For state preparation, the corresponding coefficient nonzero position values are also recorded and located in the two-dimensional *YX* position using the rest 8 numbers of qubits shown at the bottom of Fig. 2. After that, convert them into a binary system and count how many times one’s happened in one coefficient. The quantum circuits only deal with only ones and zeros that are governed by the qubits. The corresponding position value of each coefficient is used to prepare the quantum state. There were two kinds of bit rates; one is for the coefficient and another for its position.

**Figure 3:**
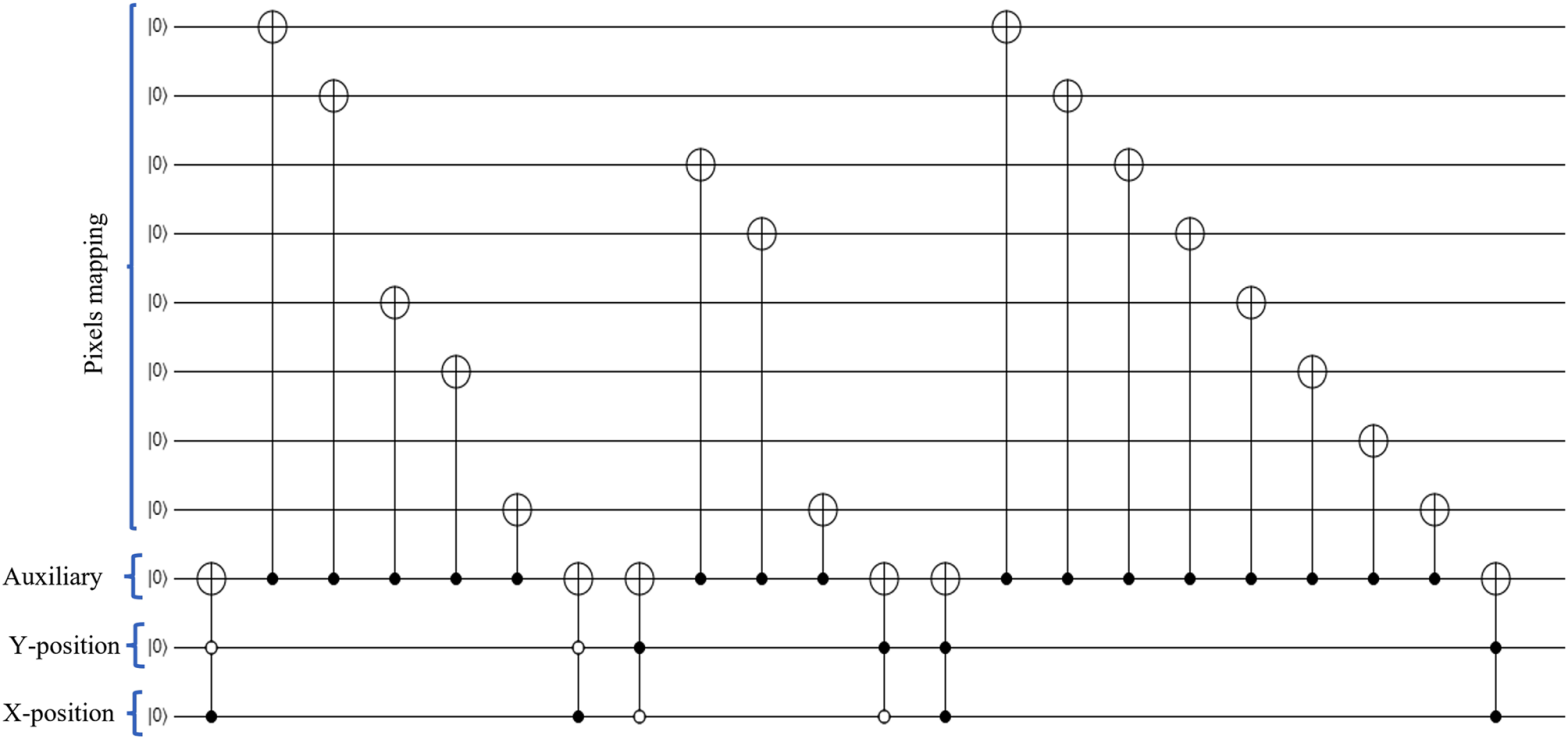
Example of decomposition circuit using Toffoli gate.

Polar coordinate-based quantum image presentation was proposed and known as QUALPI^31^. A multidimensional representation of the color image, known as a NASS(normal arbitrary superposition state), was presented in^32^. In 2021, EFRQI (Efficient Flexible Representations of Quantum Image) proposed to minimize the state preparation bits of the GQIR approach^16^. while the NEQR approach directly connects the pixel values to each pixel state value but the EFRQI approach connects the pixel values to the state values representing qubits via auxiliary qubits using the Toffoli gate^16^. An efficient decomposition approach based on the Toffoli gate is used by Naser et al. in^16^. For example, the below Fig. 3 shows the circuit diagram of the decomposition scheme where two Toffoli gates are used to complete each pixel connection of the gray scale images whose pixel values are 0, 70, 120 and 255 respectively.

However, an EFRQI has the following issue.

- Rather than decreasing the quantum resources, it increases the number of the required gate compared to the GQIR approach.
- Too much complexity to represent every pixel of medium or big size images such as $512\times 512$ and $1024\times 1024$, respectively.

## Proposed methodology

**Figure 4:**
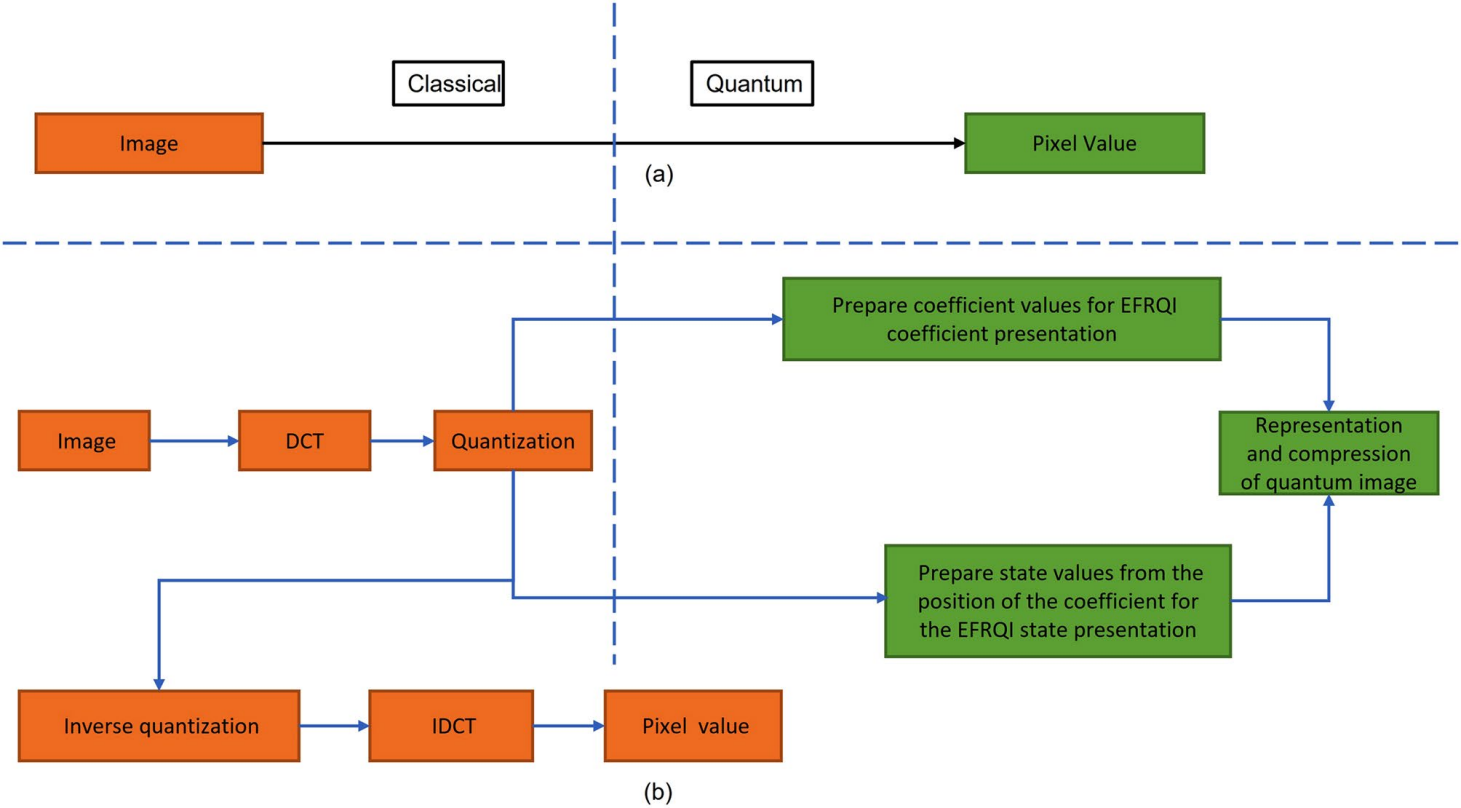
(**a**) Traditional scheme and (**b**) Proposed scheme.

In this section, the methodology of the proposed approach is described. Figure 4a shows the conventional scheme that converts the pixel value directly into the quantum domain. Figure 4b shows the overall methodology of the proposed approach. In Fig. 4, the Orange colored block indicates the classical computation whereas the green indicates the quantum. Compared to the traditional scheme, the proposed scheme deals with coefficient transform rather than representing the pixel value directly, which is done via the classical preparation approach. After that, the quantized coefficient value and its corresponding position are prepared to make it suitable for quantum representation. These things happen in quantum computers, but operation calculation is done via classical computers. In a pre-processing step, 8 and 64 block sizes of DWT and 8 block sizes of DCT were considered, including Q = 8, 16, 32, 36, and 70 quantization factors in comparing the performance of our proposed method.

**Figure 5:**
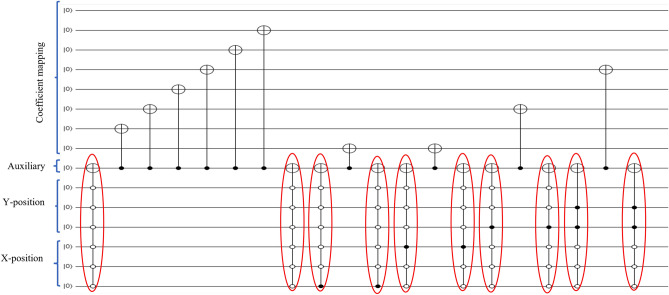
A $8\times 8$ DCT-EFRQI quantum image and its quantum state.

As an example, Fig. 5 exhibits the corresponding quantum image of deer image shown whose coefficient values are $79(X=1, Y=1)$,$10(X=1, Y=2)$, $25(X=2, Y=1)$, and $37(X=2, Y=2)$ respectively using the proposed DCT-EFRQI scheme.

In order to decrease the complexity of preparation, compression is taken via DCT transform rather than representing pixel value directly into the quantum computer to decrease the complexity of preparation. Block-wise quantum representation of coefficient is represented in quantum computer to ease the complexity of preparation. A $2^m \times 2^n$ array of images is considered for representation and compression purposes. Where *m* and *n* are the corresponding image row and column size respectively. The improvement is done through the architecture of quantum state preparation. Rather than connecting the transform coefficient directly to the state representing qubits, the connection is bypassed through the ancillary connecting qubits. The auxiliary qubit connects the transform coefficient representing qubits to the state connection representing qubits via the Toffoli gates.

In the DCT-EFRQI approach, the steps involved are given below:

- Step 1: DCT and quantize.
- Step 2: Prepare the quantized DCT coefficient and make it suitable for representing in the quantum circuit. This process is similar to the GQIR approach. The DCT-EFRQI approach requires a $q+2n+1$ number of qubits. Initially, it sets all qubits values to $\vert 0\rangle$. Where *q* is the number of required qubits calculated from the maximum value of coefficient values of the gray image after quantization. After that, *n* is calculated from the image size, for example, $n=log_2S$, where $S=S_x+S_y$ is the block size of a square image. Where $S_x$ and $S_y$ are computed from the X and Y-block sizes of the transform coefficient. One auxiliary qubit acts as a bridging qubit that connects coefficient preparation qubits and their corresponding position qubits. The initial state can be explained using the below equation^16^:
  1 $$\begin{aligned} \vert \Psi _0\rangle ={\vert 0\rangle }^{\otimes (q+2n+1)} \end{aligned}$$

Then, $(q+1)$ the identity gates and 2*n* Hadamard gates are used for coefficient preparation and its state preparation, respectively. The identity and Hadamard matrix is shown below:

2 $$\begin{aligned} I= \begin{bmatrix} 1 &{} 0 \\ 0 &{} 1 \end{bmatrix} \end{aligned}$$

3 $$\begin{aligned} H= \begin{bmatrix} 1/\sqrt{2} &{} 1/\sqrt{2} \\ 1/\sqrt{2} &{} -1/\sqrt{2} \end{bmatrix} \end{aligned}$$

In this step, the whole quantum step can be expressed as follows:

4 $$\begin{aligned} U=I^{\otimes {q+1}}\otimes H^{\otimes {2n}} \end{aligned}$$

*U* transform $\Psi _0$ from initial state to intermediate state $\psi _1$.

5 $$\begin{aligned} \Psi _1=U(|\Psi _0\rangle )=(I|0\rangle )^{\otimes {q+1}}\otimes (H|0\rangle )^{\otimes {2n}} \end{aligned}$$

The final preparation step is done using the $U_2$ quantum operator:

6 $$\begin{aligned} \Psi _2=U_2(|\Psi _1\rangle )=\frac{1}{2^n} \sum _{i=1}^{2n-1}\sum _{j=1}^{2n-1}\,|C_{YX}\rangle |YX\rangle \end{aligned}$$

where $|C_{YX}\rangle$ is corresponding coefficient value and *YX* its coordinate position. The quantum transform operator is $U_2$ is given below:

7 $$\begin{aligned} U_2=\prod _{X=0, \ldots ,2^n-1}\prod _{Y=0, \ldots ,2^n-1}\, U_{YX} \end{aligned}$$

The quantum sub-operator $U_{YX}$ is also given below:

8 $$\begin{aligned} U_{YX}= \left( I\otimes \sum _{ij\ne YX} {|ji\rangle {\langle ji|}} \right) +\sigma _{YX} \otimes |YX\rangle {\langle YX|} \end{aligned}$$

The $\sigma _{YX}$ is given below:

9 $$\begin{aligned} \sigma _{YX} =\otimes ^{q-1}_{i=0}{\sigma ^i_{YX}} \end{aligned}$$

The function of $\sigma ^i_{YX}$ sets the value of *i*th qubit of (*YX*)’s quantized DCT coefficient.

For state preparation, the Hadamard gate is used to create the superposition, and c-not is used to generate the entanglement between qubits in the quantum circuit. The identity gates do not have any effect on the qubit’s initial state which means the original state of qubits remains unchanged. The Hadamard gate create the superposition of a state $|0\rangle$ and $|1\rangle$ with equal probability.

- Step 3: Store the quantized coefficient after performing $8\times 8$ block DCT. Then, prepare the nonzero quantized coefficient for representation in the quantum system. In the meantime, record the corresponding nonzero coefficient position for preparing its quantum state. Calculate the required bits from the nonzero coefficient, which includes one’s only. In addition, when calculating its position, consider both frequent numbers of zeros and one’s happen in state connection by considering an extra bit to locate the block position that minimizes the block position error. In addition, a sign bit is also considered to assign coefficient sign position.
- Step 4: Inverse quantization.
- Step 5: Inverse DCT.
- Step 6: Compute PSNR to qualify the reconstructed image. The PSNR is defined as follows^23^:
  10 $$\begin{aligned} PSNR=20*log_{10}\frac{MAX1}{\sqrt{MSE}} \end{aligned}$$
  where *MAX*1 is the maximum possible coefficient value of an image. The MSE(Mean Square Error) is expressed as follows:
  11 $$\begin{aligned} MSE=\frac{1}{mn}\sum _{0}^{m-1} \sum _{0}^{n-1} ||(i,j)-g(i,j)||^2 \end{aligned}$$
  In the meantime, two times compression already happened through the quantum image presentation and compression. The first compression occurs in the preparation stage. Finally, it happens again when its representation in the quantum circuit since it considers only one’s and discards all zero values to prepare the coefficient.

## Result and discussion

Four image types are shown in Fig. 11 are selected from the database sample for result verification. The detail of each image is given in Table 1.

**Table 1:** Selected sample image details.

Image name	Image size
Deer	$1024\times 1024$
Cameraman	$192\times 192$
Scenery	$512\times 512$
Airport	$1024\times 1024$

**Figure 6:**
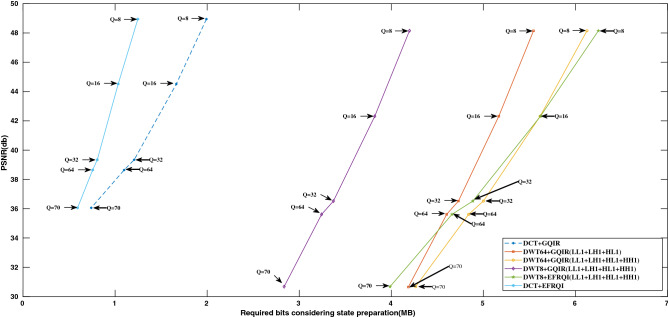
Required bits (MB) versus PSNR (db) for deer image.

Figure 6 shows the rate-distortion curve for the deer image depicted in Fig. 11a. The result shows five different quantization factor types, Q = 8, 16, 32, 36, and 70 for DCT-GQIR, DCT-EFRQI, DWT-EFRQI, and DWT-GQIR respectively. The X-axis shows the required numbers of bits in both DCT-GQIR and DWT-GQIR cases whereas the Y-axis display corresponding PSNR values for the deer image in term of the quantum domain. For both DCT-GQIR and DCT-EFRQI approaches, the $8\times 8$ block size of DCT is considered to examine each approach’s performance inside the quantum processor. On the other hand, in terms of DWT-GQIR, 8 and 64 block sizes are considered with different combinations of the label such as LL1–LH1–HL1, LL1–LH1–HL1–HH1. In the case of DWT-EFRQI, 8 block size with the label LL1–LH1–HL1–HH1 is considered as an experiment to examine how it performs inside the quantum domain. Between DWT-EFRQI and DWT-GQIR, comparison result shows that 8 DWT-EFRQI with label LL1–LH1–HL1–HH1 exhibit a better result than 8, 64 DWT-GQIR considering LL1–LH1–HL1–HH1 and LL1–LH1–HL1 label separately. This result is expected because label LL1–LH1–HL1–HH1 covers more coefficient region than label LL1–LH1–HL1 after performing DWT. In every case, it exhibits the same PSNR but different bits, although a different combination of wavelet label 1 is applied.

For example, Tables 2 and 3 show coefficient values of an $8\times 8$ array of airport images after performing DCT and DWT transformation and 8 quantization factors. In Table 3, italics, underline, bolditalics, and bold indicate LL1, HL1, LH1, and HH1 respectively where 1 indicates level 1 wavelet decomposition. The comparison table shows that DCT can generate fewer non-zero coefficients compared to DWT transformation. That’s why the DCT-based approach requires fewer qubits for state connections compared to the DWT-based approach. Some non-zero coefficients are also found in the higher state position in the case of DWT compared to DCT. Encoding the higher state position value requires more operational gates to complete the circuit connections. Thus, the DCT can reduce bits and has more capacity to concentrate low-frequency data in the top left corners so that it can represent an image with a lower number of qubits. On the hand, the DWT cannot separate the low-frequency information efficiently compared to DCT. Besides, EFRQI represents each coefficient compactly compared with the GQIR due to the use of the Toffoli gate and auxiliary qubit rather than connecting the direct approach. Thus, for given quantization factors the image loses more information in DWT compared to DCT and the results become lower quality. From the above analysis, it is concluded that DCT-EFRQI performs better compared to the DWT-GQIR approach.

**Table 2:** Coefficient of airport image after performing DCT and 8 quantization factor.

99	22	16	2	6	4	2	0
1	0	3	0	4	0	0	0
1	1	1	0	2	0	0	0
2	0	1	0	0	0	0	0
1	1	2	0	0	0	0	0
0	2	0	0	0	0	0	0
0	0	0	0	0	0	0	0
0	5	5	5	0	5	0	0

**Table 3:** Coefficient of airport image after performing DWT and 8 quantization factor.

*29*	*31*	*26*	*13*	3	2	2	0
*29*	*32*	*28*	*14*	1	2	5	1
*29*	*29*	*26*	*13*	3	1	6	1
*29*	*28*	*26*	*14*	0	1	2	0
***0***	***3***	***6***	***0***	**1**	**1**	**0**	**0**
***0***	***3***	***7***	***0***	**1**	**1**	**0**	**0**
***1***	***2***	***5***	***0***	**1**	**1**	**1**	**0**
***2***	***0***	***3***	***2***	**0**	**0**	**0**	**1**

Compared to DCT-GQIR, 8, 64 DWT-GQIR (LL1–LH1–HL1–HH1) and 8 DWT-GQIR (LL1–LH1–HL1) show poor results over considered quantization factors. It happens because DWT generates the lower coefficient value in the higher state position. As a result, preparing state preparation connection requires a higher number of bits which makes it poor performance. On the other hand, a DCT generates a higher coefficient value in the lower position. As a result, a smaller amount of state preparation bits is required to complete the state connection and make most of the coefficient zero which is discarded in the higher state position. A similar thing happens all over the coefficient region, for this reason, DCT-GQIR showed better results than DWT-GQIR.

Figure 6 also shows the proposed DCT-EFRQI scheme computational result and compares the result with the DCT-GQIR approach. The comparison result shows that the DCT-EFRQI approach shows better results compared to the DCT-GQIR approach. The result is expected because DCT-EFRQI is able to create higher coefficient values in the lower state position with the help of auxiliary qubits and the Toffoli gate. Auxilary qubit connected the coefficient values to their corresponding position via the Toffoli gate. In DCT-EFRQI, for each coefficient preparation, rather than directly connecting every coefficient one’s value representing qubits (coefficient mapping) to its state representing qubits (XY-position), it connected via the auxiliary qubits using Toffoli gate which contributes to the lower bits compared to DCT-GQIR. Moreover, the lower required bits indicated how much quantum operation resources are required to do the whole operation inside the quantum domain which defined the complexity of preparation. In the case of DCT-GQIR, for preparing for each coefficient value, it connects the coefficient representing qubits directly with its state representing qubits leading to the higher requirements of operational gates to make a complete connection which makes its poor performance. Therefore, through the rest of the result analysis, both DCT-EFRQI and DCT-GQIR approaches are considered to determine each performance using the remaining sample of images.

**Figure 7:**
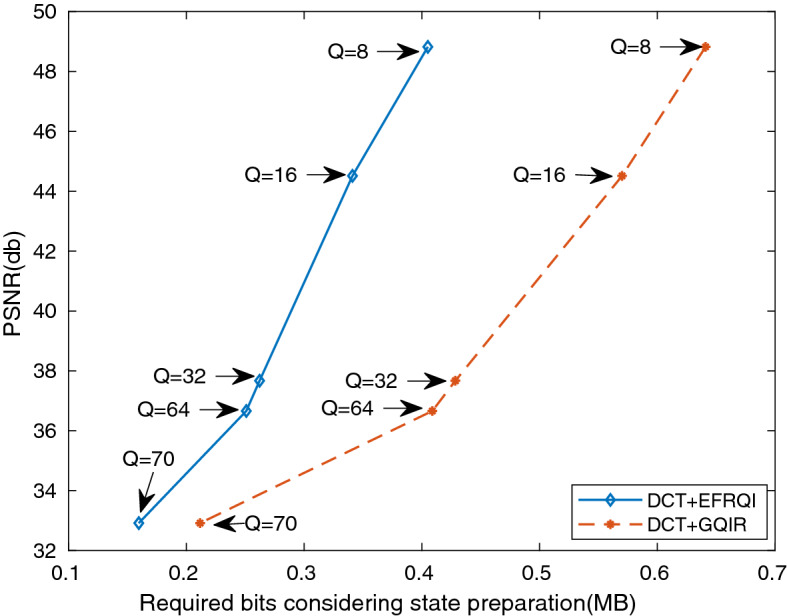
Required bits (MB) versus PSNR (db) for cameraman image.

Figure 7 shows the required bits versus PSNR result of the cameraman image for both DCT-GQIR and DCT-EFRQI approaches. This result shows how the proposed scheme is performed in case of lower resolution images such as cameraman ($192\times 192$) over considered quantization factors. The comparison result shows that the DCT-EFRQI approach shows much better results than DCT-GQIR over Q = 8, 16, 32, 36, and 70 quantization factors. The required bits make this difference, while both DCT-GQIR and DCT-EFRQI exhibit the same PSNR. Basically, after performing the DCT approach, an EFRQI approach maps the non-zero coefficient values using 17 qubits. In the case of the DCT-GQIR approach, GQIR uses 16 qubits according to its logarithmic rule of principle for state preparation connection only after performing DCT. These 16 qubits basically make the difference for compression inside the quantum domain. From the analysis of the result, It is concluded that the proposed DCT-EFRQI approach performs better in the case of low-resolution images also than the DCT-GQIR approach.

**Figure 8:**
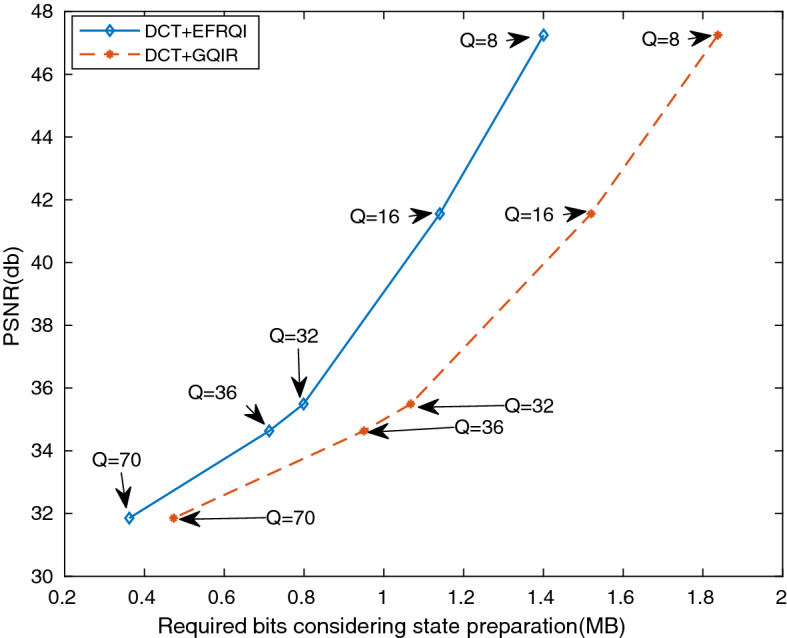
Required bits (MB) versus PSNR (db) for scenery image.

Figure 8 shows the comparison result of PSNR versus required bits for scenery images. This computational result demonstrated how the proposed scheme performs for medium size images such as scenery $(512\times 512)$ for representation and compression purposes. The required bits define how much numbers of operational gates are required to represent an image inside the quantum domain. In the proposed DCT-EFRQI approach, the 8$\times$8 DCT block is used as a preparation approach because it performs better results compared to the other block sizes. The maximum DCT coefficient value of a block of a grayscale image is 1999. After performing the quantization (e.g., the smallest value is 8), the maximum coefficient value becomes less than 255. Therefore, to represent this amount of coefficient values, 8 qubits are used for mapping. For encoding the XY position of each coefficient within a block, 8 qubits are also required to encode the state (position) of the XY-position. One auxiliary qubit is also needed to make the connection between the coefficient representing qubits and the state (position) representing qubits. In the circuit diagram, qubits is a wire which propagates from left to right. Every intersection in the Qubit connection is known as a bit which is formed by the zero, one, and c-not gates. The proposed DCT-EFRQI approach requires fewer operational resources (i.e., the required number of bits) compared to the DCT-GQIR approach. The total number of required bits differs from image to image although the image size is the same. Basically, the required number of bits makes this difference, not qubits.

While the coefficient preparation is the same in both DCT-EFRQI and DCT-GQIR approaches but the difference is found in the state preparation method. The state preparation method makes the difference between DCT-EFRQI and DCT-GQIR approaches for representation. On the other hand, quantization basically performs the compression. The comparison result indicates that the DCT-EFRQI approach provides better-required bits than DCT-GQIR but exhibits the same PSNR over Q = 8, 16, 32, 64, and 70 quantization factors. Although the same preparation approach is applied in both DCT-EFRQI and DCT-GQRI cases, the EFRQI represent method makes this different using 8 qubits with the help of auxiliary qubits and Toffoli gate for state preparation connection. From this result, It is concluded that for medium size images, the DCT-EFRQI enacts an efficient compression method compared to DCT-GQIR for quantization factors, Q = 8, 16, 32, 36, and 70 respectively.

**Figure 9:**
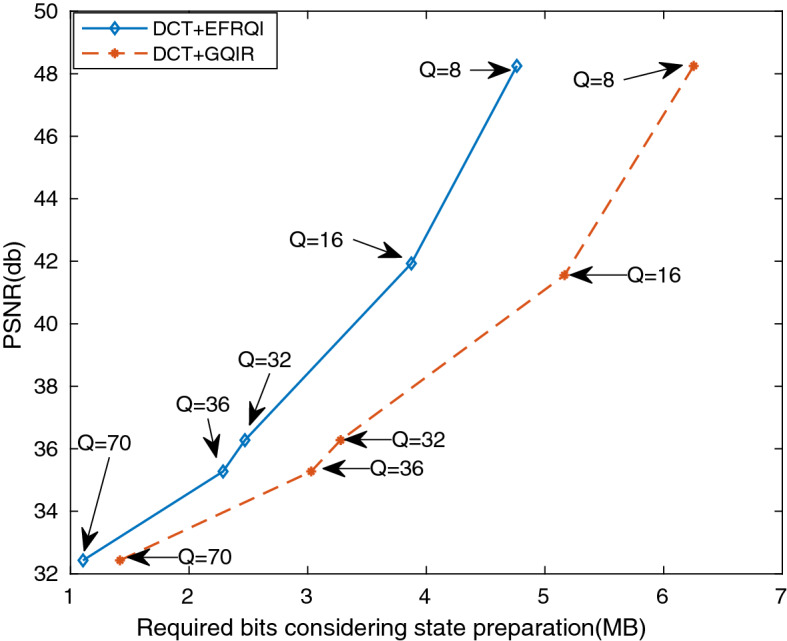
Required bits (MB) versus PSNR (db) for airport image.

Figure 9 reveals the result of the bit rate versus PNSR of airport image for both DCT-EFRQI and DCT-GQIR approaches. This result demonstrates how the proposed scheme performs for the representation and compression of the big-size image such as Airport ($1024\times 1024$) compared to the DCT-EFRQI approach over considered quantization factors. For each quantization factor, the comparison result shows that the proposed DCT-EFRQI approach represents and compresses the big-size image in a more compressed way compared to DCT-GQIR. That means the number of operations required gates is less compared to the DCT-GQIR approach. Therefore, DCT-EFRQI provides a better result in the cases of 8, 16, 32, and 70 quantization factors in terms of the required bits for big-size images also. On the other hand, in terms of PSNR, a similar quantization factor provides the same PSNR value which means PSNR does not have any effect on the rate-distortion curve. The result is expected since a similar connection is happening through the whole image coefficient. From this result analysis, it is concluded that DCT-EFRQI display better results than the DCT-GQIR approach in term of required bits but is similar in the case of PSNR.

**Figure 10:**
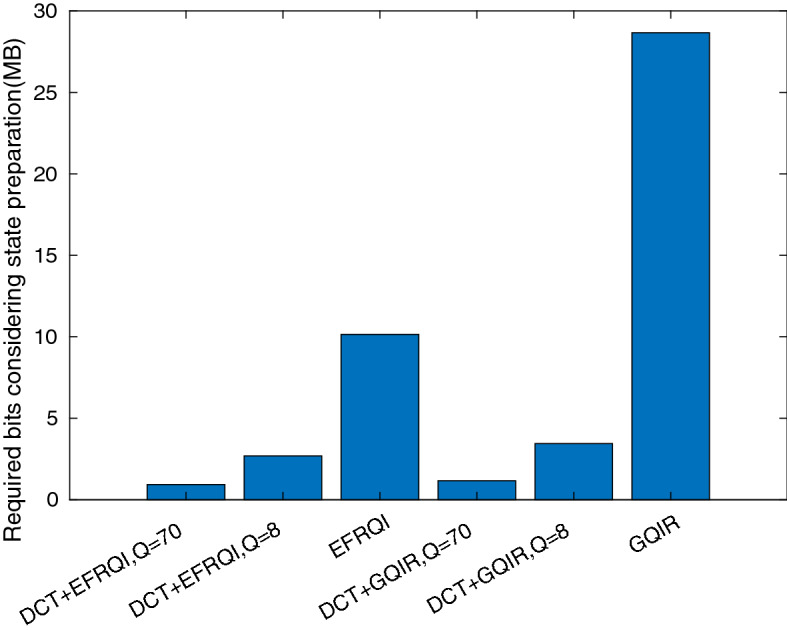
Required bits comparison for deer image.

Figure 10 shows the required bits comparison result of our proposed in the case of a deer image to determine the proposed DCT-EFRQI approach performance method compared to EFRQI, GQIR, and DCT-GQIR approaches respectively. In both DCT-EFRQI, and DCT-GQIR approaches, the quantization factors (QF) = 8, 70 are used. The comparison result shows that the GQIR requires a higher required bit compared to all others. Compression ratios usually vary based on choosing quantization factor, image, and algorithm workflow. For QF = 70, the DCT-EFRQI compression ratio is 10.8985:1 compared to EFRQI. When, QF = 8, the 3.7812:1 compared to the direct EFRQI approach. The compression ratio shows that more compression has happened in the case of the QF = 70 DCT-FRQI approach than in EFRQI. On the other hand, in terms of DCT-GQIR, DCT-GQIR shows better results for QF = 70 compared to QF = 8. The compression ratio of DCT-GQIR, for both QF = 70, and 8 is 24.611:1, and 8.3255:1 respectively compared to the GQIR approach. From this result analysis, it is concluded that the DCT-EFRQI performs more compression compared to EFRQI, GQIR, and DCTGQIR respectively.

**Figure 11:**
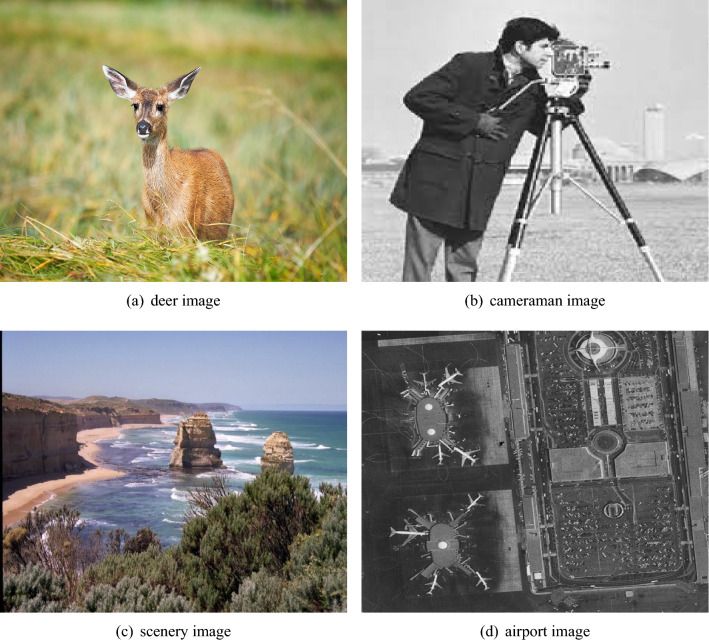
Sample image from database^33,34^.

Table 4 shows the comparison results of the compression ratio of the EFRQI scheme with GQIR without considering the preparation step. The compression ratio is the ratio of an image size before and after performing compression. In quantum computing, the compression ratio is the ratio between the required number of operational gates before and after compression happens. The required number of gates defines the complexity of the quantum circuit for image representation and compression. The cost of the quantum circuit depends on its circuit complexity. To lower the quantum circuit implementation cost, there is no alternative to decrease its complexity by means of minimizing the required operational gates. In such cases, compression is the main factor in achieving the cost reduction goal of quantum circuit connection.

In DCT-EFRQI, for one coefficient connection, it only uses two Toffoli gates to complete each coefficient connection. In the DCT-EFRQI approach, only 8 qubits (i.e., 4 qubits for the X-position and 4 qubits for the Y-position) are used to prepare the state connection. In the case of the DCT-GQIR approach, the number of required Qubits for state connection depends on image size. For example, a $512\times 512$ array of an image requires 18 qubits (i.e., 9 qubits for the X-position and 9 qubits for the Y-position) to map the state connection leads the more complexity compared to the DCT-EFRQI approach. In addition, to make a circuit connection for each one’s value occurred in each coefficient, every time it directly connects the coefficient representing qubits to the position representing qubits separately. The frequent number of ones inside each coefficient requires more operational gates to complete the circuit connection. In such a way, to complete a circuit connection to represent the whole image, the DCT-GQIR requires a more operational gate compared to the DCT-EFRQI approach.

Four types of images have been tested as stated in Table 4 without considering quantization. In the case of the Deer image, the EFRQI draws a higher compression ratio (3.2033:1) than GQIR (1.1339:1). For the cameraman image, the compression of EFRQI is 2.1623 times than GQIR. For the Scenery image, the EFRQI and GQIR provide compression ratios are 3.2485:1, and 1.1617:1 respectively. For airport images, the compression ratio of the EFRQI approach is 3.1360 compared to the GQIR approach. The comparison result shows that the EFRQI approach provides much better compression in the case of higher-resolution images than in lower-resolution. From this result analysis from the compression point of view, the EFRQI approach provides better for all cases of considered images than the GQIR approach.

**Table 4:** Compression ratio comparison without considering preparation step.

Image name	Compression ratio		The number of operation after/before compression	
	EFRQI	GQIR	EFRQI	GQIR
Deer	3.2033:1	1.1339:1	10,145,000/ 32,497,541	28,659,610/32,497,541
Cameraman	2.5864:1	1.1961:1	371,300/960,324	802,903/960,324
Scenery	3.2485:1	1.1617:1	2,327,300/7,560,121	6,507,890/7,560,121
Airport	3.6319:1	1.1581:1	8,950,100/32,505,481	28,067,709/32,505,481

Table 5 compares the compression ratio of the proposed DCT-EFRQI scheme with DCT-GQIR for all considered images using 8 and 70 quantization factors via the DCT preparation approach. The two quantization factors 8, and 70 are considered to demonstrate DCT-EFRQI and DCT-GQIR approaches capability. In the case of the Deer image, for 8 and 70 quantization factors, the compression ratio of DCTEFRQI and DCTGQIR are 12.1115:1, and 9.4401:1; 34.9083:1, 27.9069:1 respectively. For cameraman image, the DCTEFRQI exhibit more compression than DCT-GQIR. The compression ratio of the Scenery image is higher than DCT-GQIR. In terms of airport image, still, the DCT-EFRQI approach shows better capability compared to DCT-GQIR. From this analysis of the result, it is seen that a higher compression ratio is found in higher resolution images than medium and lower size images. On the other hand, the DCT-GQIR complexity is too high compared to the proposed DCT-EFRQI approach. In addition, in between DCT-EFRQI and EFRQI, a comparison of compression ratio from Tables 4 and 5 conclude that the DCT-EFRQI has superior compression capability compared to EFRQI also.

**Table 5:** Compression ratio comparison including preparation step.

Image name	Compression ratio		The number of operation after/before compression		Quantization factor (QF)
	DCTEFRQI	DCTGQIR	DCTEFRQI	DCTGQIR	
Deer	12.1115:1	9.4401:1	2,683,200/ 32,497,541	3,442,500/32,497,541	8
	34.9083:1	27.9069:1	930,940/32,497,541	1,164,500/32,497,541	70
Cameraman	8.4543:1	6.8258:1	113,590/960,324	140,690/960,324	8
	18.1993	13.8785:1	52,767/960,324	69,195/960,324	70
Scenery	8.0035:1	6.1766:1	944,600/7,560,121	1,224,000/7,560,121	8
	25.8254:1	19.9581:1	292,740/7,560,121	378,800/7,560,121	70
Airport	6.8250:1	5.1978:1	4,762,700/32,505,481	6,253,700/32,505,481	8
	29.3768:1	22.9235:1	1,106,500/32,505,481	1,418,000/32,505,481	70

## Conclusion

This paper focuses on quantum image representation and compression and proposes a new quantum image compression scheme. From this work, the below things are concluded compared to the previous method^16,23^:

- Any size of the image can be represented and compressed in the quantum system.
- Provides better required bits.
- PNSR does not have any effect if no transform or combination is happening inside the quantum system.
- Required Bits is the main fact to represent the image inside a quantum computer
- It’s simple and fast for calculating quantum operation resources.
- It opens up a lot of opportunities for processing and compressing the classical image inside the quantum processor.
- For medium-size images, DCTEFRQI compresses more than two times compared to high-size images.

## Data Availability

All data gathered or analyzed during this study are included in this published article.
